# No evidence of improvements in inhibitory control with tRNS

**DOI:** 10.1016/j.ynirp.2021.100056

**Published:** 2021-10-01

**Authors:** Etienne Sallard, Ethan R. Buch, Leonardo G. Cohen, Romain Quentin

**Affiliations:** a Human Cortical Physiology and Neurorehabilitation Section, National Institutes of Neurological Disorders and Stroke, National Institutes of Health, Bethesda, MD, USA; b Institute of Psychology, Brain Electrophysiology Attention Movement Laboratory, University of Lausanne, Switzerland; c Lyon Neuroscience Research Center (CRNL), INSERM, CNRS, Université Claude Bernard Lyon 1, Lyon, France

**Keywords:** Inhibitory control, Inferior frontal gyrus, Non-invasive brain stimulation, Magnetoencephalography

## Abstract

Previous work suggests that transcranial random noise stimulation (tRNS) over the prefrontal cortex could influence inhibitory control. Nevertheless, the specific neural mechanisms underlying this proposed effect have not been investigated. Here, we aimed at exploring behavioral and neurophysiological effects of tRNS applied over bilateral inferior frontal gyrus (IFG) on inhibitory control performance during a Go/No-go task (GNG). Nineteen participants performed two tRNS sessions (real and sham) in a double-blind crossover design study. Stimulation was applied over the bilateral IFG during 10 min at 1 mA (range: 1/+1). Resting-state MEG activity was recorded before and after tRNS, and performance on a GNG task was tested after tRNS. Behavioral performance during the GNG task was measured by the false alarm rate (FA), the reaction time in Go (RT Go) and No-go trials (RT FA), and the variability in Go responses (individual coefficient of variability, ICV). Neurophysiological impact of tRNS was assessed using global field power and power spectral density analysis of the MEG recording during the GNG task and the resting-state, respectively. tRNS stimulation did not impact inhibitory control performance. MEG analysis showed significant brain modulations during the resting-state with an increase in power spectral amplitude in beta band (20 Hz) following application of real tRNS. Our results suggest that inhibitory control performance is not modulated by tRNS over the IFG. However, we observed significant modulation of oscillatory brain activity during the resting-state, suggesting that tRNS over bilateral IFG specifically targets the dominant frequency band involved in frontal cortical interactions.

## Highlights

• TRNS was applied over bilateral inferior frontal gyrus.
• MEG was recorded during inhibitory control and resting state.
• No tRNS effect has been reported in inhibitory control.
• Beta power was higher after tRNS application during resting state.

## 1 Introduction

Inhibitory control is a crucial component of executive functions because of its major involvement in daily life. Defined as the ability to suppress thoughts or motor responses (Aron, 2007; Zheng et al., 2008), inhibitory performance is measured using the Go/No-go (GNG) behavioral paradigm. In this task, participants must respond as fast as possible to a Go stimulus and to withhold responses to a No-go stimulus. The ability to suppress a prepared action (i.e., action restraint) is indexed by the reaction times (RT) during Go stimuli and the false alarm (FA) rate during No-go stimuli. Inhibitory control engages a right lateralized front-basal ganglia brain network that includes the inferior frontal gyrus (IFG), the pre-supplementary motor area and the subthalamic nucleus (Aron et al., 2007; Chambers et al., 2009). Consistently, withholding motor responses is associated with increased right IFG activity (Aron et al., 2014; Aron and Poldrack, 2006; Chikazoe, 2010; Chikazoe et al., 2009; Rubia et al., 2003). Lesion studies further supported the role of the right IFG in inhibitory control (Aron et al., 2003; Chambers et al, 2006, 2007; Floden and Stuss, 2006; Picton et al., 2007). For instance, patients with right IFG damage showed inhibitory control deficits highlighted by more variable Go response RTs during a Go/No-go task (Picton et al., 2007).

Interestingly, applying transcranial electrical stimulation (tES) such as direct current stimulation (tDCS) might modify inhibitory control performance by increasing or decreasing neural excitability via anodal or cathodal stimulation, respectively. For instance, the improvement of inhibitory control has been observed during a Stop-Signal Task (SST: consist to respond as quick as possible to go stimuli and withhold responses when a stop signal replaces a go stimulus) after applying anodal tDCS over the right IFG as measured by a stop-signal reaction time reduction (Ditye et al., 2012; Jacobson et al., 2011). Another tES method, transcranial alternating stimulation (tACS), consists of the application of oscillating electrical current to entrain endogenous brain oscillations. tACS has been shown in numerous studies to improve measures of cognitive performance (Klink et al., 2020). However, no effect on inhibitory control performance was observed for 6-Hz theta tACS applied over the right IFG during and after a GNG task (Brauer et al., 2018). Finally, the transcranial random noise stimulation (tRNS; Terney et al., 2008), where an alternate current with random amplitude and frequency is applied to the scalp, might be of greater interest because (a) it allows proper blinding (i.e., tRNS has higher cutaneous perception thresholds and lower detection rate compared to tDCS; see Ambrus et al., 2010), (b) has polarity-independent effects and (c) appears to elicit stronger effects than tDCS on corticospinal excitability (Inukai et al., 2016), tinnitus reduction (Vanneste et al., 2013) and visual discrimination performances (Fertonani et al., 2011) when applied over motor, temporal and visual cortical areas, respectively. To our knowledge, only two studies have investigated the effect of tRNS on inhibitory control using the GNG paradigm – with contradictory results (Brauer et al., 2018; Brevet-Aeby et al., 2019). In Brauer et al. (2018), tRNS applied at 1 mA (0 mA offset; 0.1–640 Hz) during 20 min over unilateral right IFG (return electrode over the left supraorbital area) had no effect on inhibitory control. In Brevet-Aeby et al. (2019), stimulation over the bilateral dorsolateral prefrontal cortex (DLPFC) at 2 mA (1 mA offset; 100–500 Hz) improved inhibitory control as evidenced by a significant decrease in RT without changes in FA rate. These contradictory results might come from differences in the stimulation protocol. First, high-frequency tRNS alone (i.e., 101–640 Hz) has been functionally associated with cortical excitability increased (Terney et al., 2008), making it possible that the neural modulation is more effective when the frequency of stimulation is specifically above 100 Hz. Second, compared to a baseline period, tRNS with 1 mA offset over the primary motor cortex (M1) has showed increased of cortical excitability while 0 mA offset resulted in no neuromodulation (Ho et al., 2015). However, the advantage of a positive offset is not clear since original finding and numerous other studies targeting motor, temporal or visual cortices evidenced cortical excitability increased (e.g., Chaieb et al., 2011; Moliadze et al., 2014; Moret et al., 2019; Terney et al., 2008) or behavioral enhancement (e.g., Fertonani et al., 2011; Joos et al., 2015; Vanneste et al., 2013) with a 0 mA offset. Finally, although Brevet-Aeby et al. (2019) showed an effect of tRNS when targeting the DLPFC, a more robust effect (i.e., improvement of inhibitory control performance) seems to be induced when the IFG, and not the DLPFC, is targeted with anodal tDCS during a SST (Stramaccia et al., 2015). Such differential effects that depends on the targeted brain regions is further supported by a recent meta-analysis showing a medium effect size for IFG stimulation and an overall null effect for DLPFC stimulation in GNG and SST designs using anodal tDCS (Schroeder et al., 2020). Taken together and knowing that left IFG is also related to inhibitory control (Swick et al., 2008), we hypothesized that tRNS at high-frequency with 0 mA offset over the bilateral IFG will produce a modulation of the inhibitory control performance.

Because of the novelty of tRNS and the lack of studies coupling this non-invasive electrical stimulation technique with brain recordings, the neurophysiological effect of tRNS on brain networks engaged in inhibitory control and on resting brain oscillatory activity remains poorly understood. So far, no study has coupled tRNS with functional neuroimaging of inhibitory control and only two studies have explored the effect of tRNS on resting-state using EEG (Dondé et al., 2019; Van Doren et al., 2014). While no modulation was reported when tRNS was applied over the DLPFC (Dondé et al., 2019), a trend towards an increase in theta frequency band power was observed when tRNS was applied over the auditory cortex (Van Doren et al., 2014). Importantly, the impact of tRNS on resting-state brain activity remains unknown. Here, we explored the behavioral effect of tRNS applied over bilateral IFG regions on inhibitory control performance during a GNG task and the neurophysiological effect of the stimulation on resting-state and task-related brain activity using magnetoencephalography (MEG).

## 2 Material and methods

### 2.1 Participants

Twenty-three right-handed healthy adults completed one magnetic resonance imaging (MRI) session and two tRNS-MEG sessions. The tRNS-MEG sessions consisted of recordings of resting and task-related MEG before and after real or sham tRNS applied over the bilateral IFG. Each tRNS/MEG session lasted approximately 2 h. These procedures have been reviewed and approved by the CNS Institutional Review Board at the National Institute of Neurological Disorders and Stroke. None of the participants reported a history of neurological and psychiatric disease. Each participant provided written informed consent to the study. Four participants have been excluded to the analysis due to incomplete participation, problem in task performing (i.e., color mixed) and error rate in the GNG task greater than 2 standard deviations from the mean group. A total of 19 participants (14 females; 28 years ±6; range: 22–44) were included in analyses.

### 2.2 Procedure and task

**Fig. 1:**
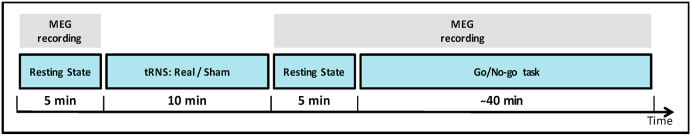
Experimental protocol progression. Real and sham tRNS sessions were pseudo-randomized in a double-blind design.

The study consisted of two separate real and sham tRNS sessions, with the session order pseudorandomized across participants. The two sessions were spaced by an interval of at least 1 week. To avoid carry-over effects of real stimulation to sham tRNS sessions for participants receiving real tRNS first. During each session, participants were comfortably seated in a shielded room in front of a screen displaying task-related information. Each tRNS/MEG session began with a resting-state MEG recording (5 min) before and after real or sham tRNS (10 min), followed by a GNG task (40 min. See Fig. 1). Both the participant and experimenter were naïve to the tRNS condition (real or sham) for all sessions (i.e., double-blind design). This blinding procedure was further strengthened by the fact that participants were unaware of the possibility of receiving sham stimulation (i.e., participants only knew that two stimulation conditions were administered but were not told how they differed).

### 2.3 Resting-state

During the resting-state, participants were required to fixate a white cross on a black background screen. A period of 5 min was recorded in each session before and after the tRNS application (pre-tRNS and post-tRNS). The time intervals between the first resting-state and the stimulation (interval-1) and between the stimulation and the second resting-state (interval-2) were controlled and not significantly different between time intervals (3 min 05 s and 2 min 38 s for interval-1 and -2, respectively) or stimulation conditions (2 min 49 s and 2 min 54 s for real and sham condition, respectively) as evidenced by a repeated-measures ANOVA (all p > 0.1).

### 2.4 Go/no-go task

The GNG task has been adapted from Hartmann et al. (2016). Participants had to respond as fast as possible to the Go stimuli by pressing a button with the right index finger and withhold their response to No-go stimuli. Stimuli were letters (A, E, M, O and S) colored in blue, green, red, white and yellow. In a given block, the No-go stimulus was either a letter or a color. All stimuli were presented on a black background at the center of the screen. Stimulus delivery and response recording were controlled using E-Prime 2.0.

A total of 10 experimental blocks of 40 trials (20 Go and 20 No-go; randomized order) were performed in each session. A calibration block of 12 trials (6 Go; 6 No-go) was performed before each experimental block. These calibration blocks enabled us to calculate the mean response time (RT) to Go trials used to determine a RT threshold (RTt) for the following experimental block. The feedback “Too late” was displayed for RTs greater than 95% of the mean RTt. This approach helped keep the same level of time pressure and motivation throughout the task for each participant (Hartmann et al., 2016; Manuel et al., 2010).

Each trial started with the presentation of a fixation cross for 1500–1900 ms, followed by one letter for 500 ms. Participants had 1000 ms to respond. Feedback was then displayed for 500 ms. The feedback consisted of a happy face for correct responses (no response to No-go trials and response to Go trials with RT < RTt), a “Too late” message for RT > RTt, an unhappy face for an incorrect response (response to No-go trials) and a “No response” message for no response to a Go stimulus within 1s. Individual responses to Go trials below 100 ms or with RT higher or lower than 2.5 standard deviation from the participant's mean RT were rejected for each block separately.

### 2.5 Transcranial random noise stimulation

tRNS was delivered by a battery-driven stimulator (DC-STIMULATOR PLUS, www.neuroconn.de) at high frequency (100–640 Hz) for 10 min (fade in/out: 15 s) at 1 mA (range +1/-1 mA; offset: 0). In the sham condition, active (i.e., 1 mA) current was delivered in the first and last 15 s of the 10-min period to facilitate blinding. The stimulation electrodes (4 cm × 4 cm) were placed in saline-soaked sponges (0.9% saline solution) and positioned over the bilateral IFG. Spatial targeting of the right and left IFG in individual participants was performed through non-linear spatial registration (FSL, FNIRT; Jenkinson et al., 2012) of the participant's T1-weighted image to the MNI-template. Based on Fan et al. (2016), a mask for the IFG was constructed in MNI-space from the Brainnetome atlas (https://pubmed.ncbi.nlm.nih.gov/27230218/) merging the BA44 dorsal (MNI: 46, 13, 24 and 45, 16, 25 for the left and right hemisphere respectively), BA44 ventral (−52, 13, 6 and 54, 14, 11 for the left and right hemisphere respectively) and the BA45 caudal (−53, 23, 11 and 54, 24, 12 for the left and the right hemisphere respectively). The inverse MNI-to-participant space registration was then computed and applied to the left and right IFG MNI-space masks, transforming the masks into each participant's original T1-weighted image space. The two stimulation target point-locations were then computed as the center of gravity for both the left and right IFG masks, respectively. The stimulation electrodes were then precisely positioned using the Brainsight neuronavigation system (v.2.2.7; Rogue Research). First, each participant's T1-weighted MRI was imported into Brainsight and used to perform a 3D reconstruction of the brain and the skin surface of the face. Second, target locations were projected onto the head surface via a surface-normal line passing through the previously defined right and left IFG targets. Third, each participant's T1-weighted image space was mapped to their scalp using standard fiducial landmarks in Brainsight. Finally, the IFG targets defined in Brainsight were used to mark electrode positions on the participant's head.

### 2.6 MRI acquisition

Magnetic Resonance Imaging data were acquired with a Siemens Skyra 3 T scanner using a 32-channel coil. High-resolution (0.93 × 0.93 × 0.9 mm^3^) 3D magnetization prepared rapid gradient echo (MPRAGE) T1-weighted images were acquired (repetition time = 1900 ms; echo time = 2.13 ms; matrix size = 256 × 256 × 192).

### 2.7 MEG recording

The magnetic field was sampled at 600 Hz with a bandwidth of 0–150 Hz using a CTF 275 MEG system (CTF Systems, Inc., Canada) composed of a whole-head array of 275 radial 1st order gradiometer/SQUID channels housed in a magnetically shielded room (Vacuumschmelze, Germany). Participants were comfortably seated inside the MEG room with their head positioned at the center of the sensor array. Head movements were recorded and controlled before and after each block with head localization coils attached over bilateral pre-auricular and nasion fiducial landmarks. When the position of the head moved more than 3 mm, a real-time head position was displayed on the screen to help participants reposition their head at its original position.

### 2.8 Behavioral analyses

Mean reaction time (RT) to the Go (RT Go) and No-go trials (RT FA), false alarm rate (FA) in No-go trials and individual coefficients of variation (ICV: [standard deviation RT Go/mean RT Go] X 100) were compared between stimulation conditions (real vs. sham) using paired *t*-test and measure of the Bayes factor. Bayes factor was computed using the Pingouin 0.3.12 Python package (Vallat, 2018). The Bayes factor (BF10) helps to determine the strength of evidence on the hypotheses (i.e., H0: null hypothesis; H1: alternative hypothesis). For instance, evidence in favor of H0 is considered as anecdotal for BF10 between 1 and 1/3, moderate for BF10 between 1/3 and 1/10 and strong for BF10 below 1/10 (Lee and Wagenmakers, 2013).

### 2.9 MEG analyses

For the GNG task, continuous data were first filtered at 1–50 Hz and then segmented in epochs of 600 ms time locked to the target (−100 ms before and +500 ms after the target) with a baseline between −100 ms and 0 ms according to the stimulus onset. A peak-to-peak MEG amplitude superior to 5e-12 T was rejected. An average of 387 ± 15 epochs in real and 385 ± 18 epochs in sham tRNS per participant were included in the analysis. The effect of stimulation on inhibitory control was assessed by analyzing the global field power (GFP) of the event-related fields (ERFs). The GFP, represented by the spatial standard deviation of the sensor values at each time point provides a quantitative measure of response-strength changes in the whole brain (Koenig et al., 2011; Koenig and Melie-Garcia, 2010). Differences in GFP were measured during the task with a data-driven, time-wise 2 × 2 ANOVA with within-subject factors stimulus (Go; No-go) and stimulation condition (real; sham) as repeated-measures.

For the resting-state, continuous data were first filtered at 1–50 Hz. Independent component analysis was performed and components corresponding to blink and heart frequency artifacts were removed after visual inspection. Then, data were segmented in epochs of 2 s with 1s overlapping. The first and last 15 s of the 5 min recording were cropped giving 270 epochs scanned by resting-state period. Epochs with a peak-to-peak MEG amplitude superior to 5e-12 T were rejected. A total of 256 ± 40 epochs in real and 263 ± 16 epochs in sham tRNS were included after artifact rejection. Power spectral density was computed from 2 to 50 Hz. Repeated-measures ANOVAs at each time sample were performed with a correction for multiple comparisons using the within-subject factors stimulation condition (real; sham) and time of stimulation (pre-tRNS; post-tRNS). Since prior studies reported weak or contradictory effects of tRNS modulation of brain activity, repeated-measures ANOVAs were also performed without correction for multiple comparison to observe trends.

## 3 Results

### 3.1 Behavior

**Fig. 2:**
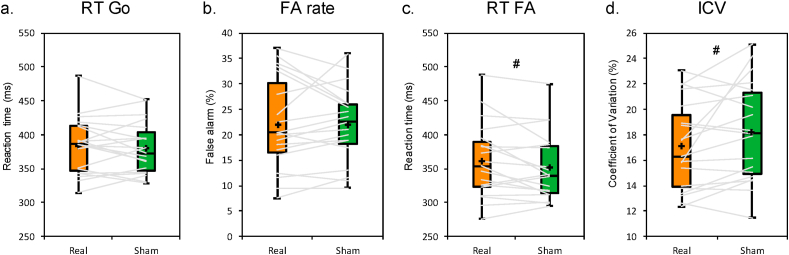
Behavioral results of the GNG task in real and sham tRNS sessions. Box plots representation of a) mean RT (ms) in Go trials, b) percentage of false alarm (FA), c) mean RT (ms) in No-go trials, and d) individual coefficient of variation (ICV) in Go trials. Horizontal gray lines represent individual participant performance in real and sham tRNS sessions. Each box plot displays the median (horizontal line), the mean (cross) and confidence intervals (Tukey whiskers).

Neither the RT Go nor the FA rate was significantly different between real and sham stimulation (t(18) = 0.52, p = 0.61, BF10 = 0.27 and t(18) = 0.09, p = 0.93, BF10 = 0.24 in RT Go and FA rate, respectively. See Fig. 2a & b). BF10 below 1/3 is considered moderate support for the null hypothesis. The mean RT FA showed a tendency with slower responses (i.e., errors) after real compared to sham stimulation (t(18) = 1.89, p = 0.07, BF10 = 1.03. See Fig. 2c). The ICV analysis revealed a tendency to lower variability after real compared to sham stimulation (t(18) = −2, p = 0.06, BF10 = 1.22. See Fig. 2d). BF10 between 1 and 3 is considered anecdotal support for the null hypothesis.

### 3.2 MEG

#### 3.2.1 Go/No-go task

**Fig. 3:**
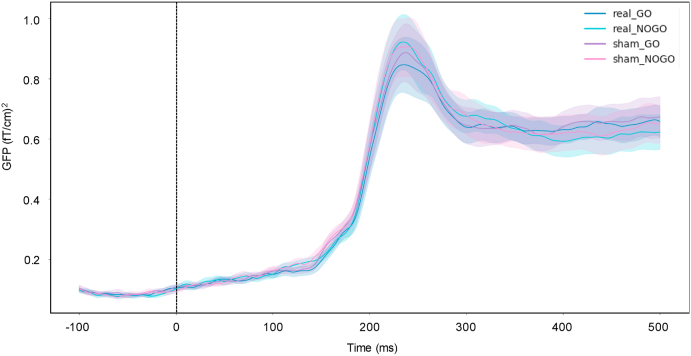
Magnetoencephalography event-related field (ERF) results of the Go/NoGo task. Global Field Power is represented for Stimulation (real; sham) and Stimulus (Go; NoGo) variables. The lines represent the GFP after real tRNS for the Go (blue line) and No-go (cyan line) trials, and after sham tRNS for the Go (purple line) and No-go (pink line) trials. The contours on each line represent the standard error of the GFP. (For interpretation of the references to color in this figure legend, the reader is referred to the Web version of this article.)

Repeated-measure ANOVA at all time samples on the GFP showed no significant difference between stimulation condition (all p > 0.05), stimulus (all p > 0.05) or interaction (all p > 0.05; see Fig. 3), with or without correction for multiple comparisons.

#### 3.2.2 Resting-state

**Fig. 4:**
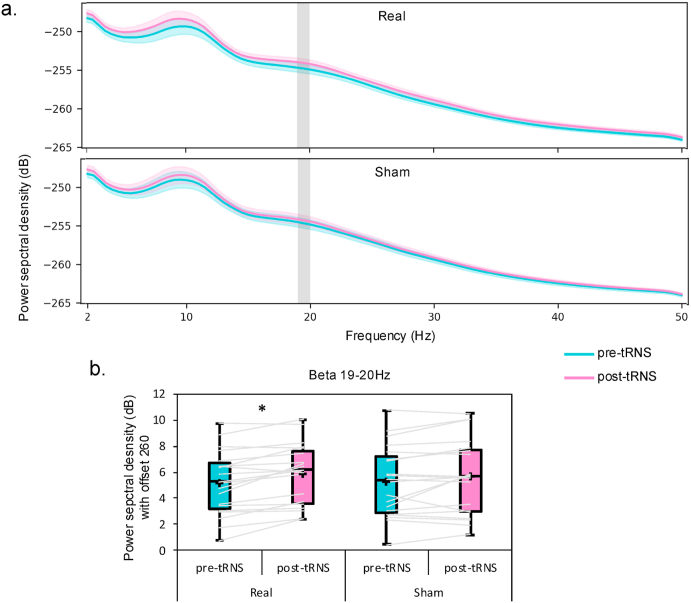
a. Power spectral density from 2 to 50 Hz during the resting-state before (pre-) and after (post-) the real and sham sessions. b. Box plots represent the 19–20 Hz interaction between time and stimulation condition. Individual participant's values (gray lines), the median (horizontal line), the mean (cross) and confidence intervals (Tukey whiskers) are represented. * corresponds to *p* < 0.05.

Repeated-measures ANOVA on the power spectral density with a correction for multiple comparisons revealed a main effect of time (cluster between 2 and 45 Hz; p = 0.01) and no effect of stimulation (p > 0.05) or interaction (p > 0.05) during the resting-state. Repeated-measures ANOVA without correction for multiple comparisons evidenced a stimulation x time of stimulation interaction in 19 (F(1; 18) = 4.95, p = 0.03), 19.5 (F(1; 18) = 4.66, p = 0.04) and 20 Hz (F(1; 18) = 4.95, p = 0.04). Power spectral density of 19, 19.5 and 20 Hz were then averaged and analyzed through a repeated-measures ANOVA confirming the stimulation × time interaction (F(1; 18) = 4.73, p < 0.05). Bonferroni post-hoc comparison revealed an increase in beta power after real tRNS but not after sham tRNS (see Fig. 4).

## 4 Discussion

In this study, we explored the behavioral and neurophysiological effects of tRNS applied over the bilateral IFG during a GNG task and resting-state neural activity. Our results found no evidence of tRNS efficacy on inhibitory control performance between real compared to sham stimulation. However, weak brain activity modulations have been identified during the resting-state after applying the real tRNS condition.

Conversely to our expectations, no evidence for influences of tRNS on inhibitory control performance was observed as revealed by similar RT (for RT Go and RT FA), percentage of FA and ICV between real and sham stimulation conditions. Our results are contradictory to Brevet-Aeby et al. (2019), who reported an improvement of inhibitory control performance after applying tRNS over the DLPFC, but in line with Brauer et al. (2018), who showed no behavioral effect after targeting the right IFG. Although these differences might be related to the targeted area, previous tDCS studies also reported null finding during a GNG task independently of the targeted area (Beeli et al., 2008; Campanella et al., 2017; Conley et al., 2015; Cunillera et al., 2016; Sallard et al., 2018). For instance, Beeli et al. (2008) observed no behavioral change after anodal stimulation and an increase in FA rate after cathodal tDCS over the right DLPFC. In Campanella et al. (2017) and Sallard et al. (2018), no inhibitory control change is reported after applying anodal tDCS over the rIFG. Additional investigations directly comparing these regions have suggested a more reliable effect of the stimulation over the IFG compared to the DLPFC as measured by GNG and SST (Schroeder et al., 2020; Stramaccia et al., 2015). Therefore, our null finding might result from another methodological difference. Specifically, the use of a DC offset as in Brevet-Aeby et al. (2019) might induce differences in stimulation reliability. It has been demonstrated that applying tRNS with a DC offset is more likely to increase cortical excitability than tRNS without a DC offset (Ho et al., 2015). Accordingly, our tRNS parameters, without a DC offset, might not be efficient enough to boost inhibitory control performance. However, this parameter might not play a key role in tRNS effect since numerous studies using a 0 mA offset evidenced neurophysiological and/or behavioral modifications (e.g., Chaieb et al., 2011; Fertonani et al., 2011; Joos et al., 2015; Moliadze et al., 2014; Moret et al., 2019; Terney et al., 2008; Vanneste et al., 2013). Finally, one last possible reason for the null finding might come from the difficulty level of the task. With a short response time to the Go trials and few inhibitions failure to the No-go trials (probably favorized by a 50/50 ratio of go and no-go stimuli), participants easily performed the task. Thus, the neuromodulation induced by tRNS might not interact with the GNG task requirements employed in this study, which may not have required sufficient inhibition. In this way, previous studies have observed an effect of the tES only on cognitive tasks with a high difficulty (e.g., for tDCS see Gill et al., 2015; for tACS see Moliadze et al., 2019; for tRNS see Popescu et al., 2016). Commonly used to measure inhibitory control performance, behavioral improvements have been repeatedly measured during the SST (e.g., Cai et al., 2016; Ditye et al., 2012; Jacobson et al., 2011) whereas no change has been reported in the GNG task (e.g., Campanella et al., 2017; Cunillera et al., 2016; Sallard et al., 2018) after applying tDCS. This task effect is further supported by a meta-analysis reporting higher effect size of tDCS on inhibitory control in SST compared to GNG task (Schroeder et al., 2020). Therefore, we suggest that inhibitory control modulation by tES might be more efficient to modulate action cancellation (i.e., in SST) than action restrain (i.e., in GNG) mechanisms.

Supporting our behavioral results, ERF-based analysis of the MEG data did not reveal brain significant changes in GFP after tRNS during the GNG task. Although no study previously attempted to investigate neural processes of inhibitory control after tRNS sessions, this result is consistent with an EEG study applying tRNS over DLPFC where neither brain activity modulations nor behavioral changes were observed during a Stroop task (Dondé et al., 2019). This null finding might be due to the use of non-optimal stimulation parameters or insufficient task difficulty as discussed above.

Analysis of power spectral density over the entire MEG sensor array during showed that real tRNS increased resting-state beta (19–20 Hz) frequency band power relative to sham. Since frontal cortex exhibits dominant oscillatory activity over a range of 21–50 Hz (Rosanova et al., 2009) and tRNS applies alternate current at random frequencies, it is possible that the dominant frequency mode associated with the frontal cortex is modulated by the stimulation. This specific link between the frequency of the modulated activity and the stimulated region is supported by Van Doren et al. (2014) where a trend toward a theta (4–7.5 Hz) power increase was observed after applying tRNS over the bilateral temporal cortex, a frequency band associated with temporal regions (Niedermeyer, 1999). It suggests that tRNS enhances the specific frequency associated with the targeted area. However, this interpretation must be taken with care since the power modulation observed in Van Doren et al. (2014) nor in our experiment reached statistical significance when assessed with robust statistical tests. The power magnitude change observed in our study was characterized by a slightly lower frequency mode than the endogenous frequency band observed in the targeted frontal region. In fact, endogenous neural oscillations within our specific beta frequency band are predominantly engaged over the sensorimotor cortex (Niedermeyer, 1999), which is located nearby our targeted area. Although effect of tRNS on functional connectivity between brain regions is not understood, it has been suggested that tDCS can induce remote effects on areas that are not directly targeted by the stimulation (Nitsche, 2011). It is then a possibility that tRNS has induced widespread neuromodulation beyond the targeted frontal region.

In summary, our study investigated the behavioral and neurophysiological effects of tRNS on inhibitory control performance and resting-state brain activity. Overall, tRNS stimulation did not influence inhibitory control performance or its neural correlates during a GNG task. However, we showed that tRNS did increase resting-state beta power in the targeted area. Future investigations using longer stimulation durations or greater stimulation insensities might result in behavioral modulations and could help to better identify neurophysiological modulations of inhibitory control induced by the stimulation.

## Declaration of competing interest

The authors declare that they have no known competing financial interests or personal relationships that could have appeared to influence the work reported in this paper.
